# Does imepitoin reduce signs of fear and anxiety in dogs frightened by loud noises?

**DOI:** 10.18849/ve.v11i3.745

**Published:** 2026-08-14

**Authors:** Hannah McNicholas

**Keywords:** ANXIETY, CANINE, DOGS, FEAR, IMEPITOIN, NOISE PHOBIA

## Abstract

**PICO question:**

In adult pet dogs with a sensitivity to sudden loud explosive noises, does treatment with imepitoin, compared to no medical treatment, reduce levels of fear and anxiety?

**Clinical bottom line:**

**Category of research:**

Treatment.

**Number and type of study designs reviewed:**

Two papers were critically reviewed. Both were double blind randomised placebo-controlled clinical trials.

**Strength of evidence:**

Moderate.

**Outcomes reported:**

Imepitoin given orally twice daily to adult dogs who were sensitive to loud noises such as fireworks or thunderstorms reduced owner rated levels of fear and anxiety responses when compared to placebo. Owner satisfaction with imepitoin treatment was mostly good, but a proportion of dogs developed mild-to-moderate side effects including ataxia, appetite changes, lethargy, and emesis during treatment.

**Conclusion:**

In adult pet dogs with a sensitivity to sudden loud noises, treatment with imepitoin, compared to no medical treatment, may reduce owner-rated levels of fear and anxiety responses in their dogs. Imepitoin appears to be generally well tolerated by dogs, is considered easy to administer by owners and can positively improve the welfare of dogs who show fear and anxiety in response to loud noises including fireworks and thunderstorms. Whilst no serious adverse effects were noted, a high prevalence of side effects were observed in the reviewed studies which were carried out using the manufacturer’s recommended dose for noise phobia. These side effects were shown to be unacceptable to some owners, resulting in reduced compliance and satisfaction with treatment. Clinicians should proactively discuss potential side effects of imepitoin with owners, particularly if used at the manufacturer's recommended dose, and consider starting at a lower twice daily dose of 10 mg/kg and titrating up to 30 mg/kg as required.

How to apply this evidence in practice

The application of evidence into practice should take into account multiple factors, not limited to: individual clinical expertise, patient’s circumstances and owners’ values, country, location or clinic where you work, the individual case in front of you, the availability of therapies and resources.

Knowledge Summaries are a resource to help reinforce or inform decision making. They do not override the responsibility or judgement of the practitioner to do what is best for the animal in their care.

## Clinical scenario

During a routine visit a client mentions to you that her normally calm 4-year-old Labrador is increasingly fearful of loud noises. They live near to farmland where regular shoots are held, and last year he became so agitated by the gunshots that she worried he might injure himself. She has tried making him a safe den to hide in and ignoring his behaviour, although this is challenging. In addition to this she has used calming diffusers, herbal tablets, flower essences, and a pressure vest, none of which made much difference. She did plan to help him using sound therapy as a vet had previously suggested but had not had chance to use it yet. With the shooting season starting again and Fireworks Night approaching, she asks whether there are any tablets she can give for a few days at a time to alleviate signs. You have used short-term alprazolam in the past for other noise phobic dogs, but wonder about the effectiveness of imepitoin, which is licensed for this problem.

## The evidence

Two prospective double-blind randomised placebo-controlled clinical trials addressed the PICO question. Both used samples of client-owned dogs who were evaluated in the home environment. Engel et al. (2019) examined using imepitoin for dogs with noise phobia who had previously reacted to firework noises, whereas Perdew et al. (2021) specifically examined imepitoin use for storm anxiety.

The strongest evidence comes from a multicentre study by Engel et al. (2019) with a sample size of 226 dogs (in full analysis set), which demonstrated a statistically significant reduction in owner-reported fear and anxiety behaviours. These behaviours, however, were assessed using a scale which had not been validated separately from this study (Mills et al., 2012). Perdew et al. (2021), a smaller study of 45 dogs (in the intention-to-treat analysis set), showed a reduction in owner-reported fear and anxiety in the imepitoin-treated dogs. The small sample size and high withdrawal rate from the imepitoin group, however, affects the validity and transferability of their results. In both studies high numbers of mild-to-moderate adverse events (side effects) in the imepitoin groups potentially led to a degree of unblinding and the introduction of bias, particularly as both studies relied on subjective owner assessment for results. Taking these limitations into consideration, alongside the low number of studies which answer the PICO question, the evidence was considered moderate.

**Engel et al. (2019):** Effectiveness of imepitoin for the control of anxiety and fear associated with noise phobia in dogs **Aim: **To determine the effectiveness and safety of imepitoin compared to placebo for reducing anxiety and fear associated with canine noise phobia.

Population:	Client-owned dogs with a history of signs of noise phobia, in mainly urban locations at 17 German and four Dutch study sites.Inclusion criteria: Dogs weighing ≥ 2 kg Dogs scoring > 30 on Lincoln Sound Sensitivity Scale (LSSS) Dogs were negative for medical causes of noise sensitivity detectable on clinical examination. Diagnosis of noise phobia was confirmed, and generalised anxiety excluded, where fear responses were reliably and specifically triggered by identifiable explosive noises (fireworks, thunderstorms, gunshots) rather than being generalised to a diffuse range of triggers. Exclusion criteria: Dogs who had received imepitoin to treat epilepsy in the previous five months. Dogs who had been treated for noise phobia in the previous 12 months. Factors affecting risk to people in the dog’s family, for example, dogs showing aggressive tendencies, including towards other dogs or people in the home.
Sample size:	251 dogs.
Intervention details:	251 dogs were block randomised using a pseudorandomised number generator. 13 were excluded due to poor treatment compliance and a further 12 were excluded due to a lack of data postrandomisation, resulting in the full analysis set (226 dogs). The full analysis set comprised two groups: treatment with imepitoin (n = 104) and placebo (n = 122). Baseline data were gathered with a questionnaire assessment of intensity of 16 behaviours during a 15 minute period four days before expected fireworks. Behaviours associated with fear such as restlessness/pacing, cowering, hiding or vocalising were classified on an ordinal scale from 0 = not present, 1 = small amount up to 5 = extensive amount. Imepitoin was administered at approximately 30 mg/kg twice daily, per os (by mouth). Placebo: visually identical placebo tablets twice daily, per os. Six doses were given at approximately 12-hour intervals starting on the morning of 29 December and ending with a dose between 18:00 and 20:00 hours on 31 December. A person well known to the dog was present that evening, and owners were advised to minimise stress by neither punishing nor reassuring if frightened, ignoring noises, and keeping the dog in a safe, secure location at home.
Study design:	Randomised placebo-controlled trial.
Outcome Studied:	Owner's overall assessment of treatment (rated from excellent effect on behaviours to worse than previously): a generalised linear model estimated the odds ratio of imepitoin versus placebo, with treatment as a fixed effect and site and treatment-by-site as random effects. For the intensity of signs of a dog's fear and anxiety (based on the cumulative scores of 16 behaviours): a mixed model was used, with time point and the time point-by-treatment interaction as fixed effects, and subject, site, and the site-by-treatment interaction as random effects. Adverse event type and prevalence.
Main Findings (relevant to PICO question):	In full analysis set (FAS): Statistically significant number of owners reported a good or excellent treatment effect in the imepitoin group compared to placebo. Odds ratio of 4.7 (95% CI,2.79–7.89), P < 0.0001. Approximately 65% of owners in the imepitoin group rated treatment as 'good' or 'excellent', compared with approximately 25% in the placebo group (estimated from Supplementary Figure 1; exact values not reported in text). Significant statistical reduction in fear and anxiety behaviours as measured on 16-item modified LSSS questionnaire in imepitoin group compared to placebo at 00:20 hours on 1 January. Difference of –6.1 scoring points, P < 0.0001. Signs of fear and anxiety were lower in imepitoin treated dogs compared to placebo at every time point. Lower owner-rated anxiety scores at 00:20 hours in imepitoin group: 16.64 (SD 11.57) compared to placebo group 24.88 (SD 13.08). Dogs treated with imepitoin returned to near baseline score at 01:00 whereas dogs in placebo group did not, P < 0.0001. 48% (55/114) of imepitoin-treated dogs experienced adverse events compared with 10% (13/124) of placebo-treated dogs, most frequently ataxia 35% (40/114) imepitoin vs 2% (2/124) placebo and increased appetite 18% (21/114) imepitoin vs 1% (1/124) placebo.
Limitations:	No power calculation regarding optimal group size is detailed in the paper. Any standardisation of external variables during baseline measurement on 27 December not specified for example time of day, before or after exercise. Dogs with a history of aggressive behaviours were excluded; the study authors suggested that further studies are required for this population. Pseudorandomisation produced different sized treatment (imepitoin n = 121) and control (placebo n = 130) groups. Constituents of placebo not detailed. Reliance on accurate dosing, and reporting of dosing, by owners. High incidence of mild-to-moderate adverse effects in imepitoin group (in total = 48%, primarily ataxia = 35%). High incidence of ataxia in treatment group 35% (40/114) compared to placebo 2% (2/124) has potential for unblinding owners and investigators to which dogs were in treatment group. Dose adjustments for dogs with ataxia in either treatment or placebo group not described. Although owners were given general advice on minimising stress, no other standardised environmental or behavioural modification advice was provided and no aspects of the home environment were controlled for. Owner’s response to dog’s fearful behaviour not known. Subjective assessment of behaviours by owners, not expert observation. Modified LSSS is used to measure only intensity of behaviours rather than intensity and frequency of behaviours as it was validated for. Recall bias comparing reactions to fireworks whilst on treatment to reactions to fireworks the previous year. No description of duration, intensity, or proximity of fireworks for either group. α-level was not stated. Study funded by Boehringer Ingelheim manufacturers of Pexion® (imepitoin) one co-author is holder of a patent related to imepitoin.

**Perdew et al. (2021):** Evaluation of Pexion ® (imepitoin) for treatment of storm anxiety in dogs: A randomised, double‐blind, placebo‐controlled trial **Aim: **To assess the efficacy of imepitoin compared to placebo for the treatment of dogs with a history of storm anxiety.

Population:	Client-owned dogs with a chronic history of storm phobia living in homes with their owners in North Carolina, USA.Inclusion criteria: Between 1 to 12 years of age. Weight between 2 kg to 50 kg. Have a history of storm anxiety for a minimum of 12 months. Dogs scored ≥ 30 on Lincoln Sound Sensitivity Scale (LSSS). Exclusion Criteria: A history of aggression. Severe systemic impairment (e.g., cardiovascular, hepatic, renal, etc.). Dogs on other psychoactive drugs (list of potential drugs not specified). If a major life change (for example house move) was planned during the study period. Adjunctive treatments not allowed including pheromones, pressure vests and behaviour modification (unless they had been in place for over four weeks and LSSS score still ≥ 30).
Sample size:	58 dogs.
Intervention details:	58 dogs were screened: following exclusions 45 dogs remained to be randomised into treatment groups in a ratio of 2:1 active (imepitoin) n = 30 to placebo n = 15. Per protocol analysis included all dogs remaining in the final week: active (imepitoin) n = 20, placebo n = 15. All dogs had a physical examination and blood tests for complete blood count, serum biochemistry. Storm logs were completed for a minimum of two storms within a period of up to 6 weeks. Active group: imepitoin 30 mg/kg twice daily, per os (by mouth) (average dose 30.4 mg/kg SD ±3.09). Placebo group: visually identical placebo tablets twice daily, per os (average dose 29.84 mg/kg SD ±2.46). Treatment period: 28 days (± 3 days).
Study design:	Randomised placebo-controlled trial.
Outcome Studied:	Scores from individual storm logs, including date, duration and intensity of storms, and intensity of behavioural signs as listed on LSSS, with change in average weekly storm log scores monitored during treatment compared to baseline. Scores from weekly online surveys considering all storms that occurred that week, rating the frequency and intensity of behavioural signs on LSSS, with change in weekly survey scores monitored during treatment compared to baseline. Ratings from end-of-study survey completed by owners about their impression of the treatment, agreement rated on a 7-point Likert scale from 'strongly agree' to 'strongly disagree.' Average storm exposure of both groups. Adverse event type and prevalence.
Main Findings (relevant to PICO question):	Weekly survey scores for intention-to treat analysis improved to a greater degree compared to baseline scores in the active (imepitoin) group P < 0.0001 compared to placebo group P < 0.003, although both groups improved. Weekly survey scores for per-protocol analysis showed a statistically significant difference between placebo and active (imepitoin) groups during week 2 (P = 0.01) and week 4 (P = 0.02). Owners reported in final questionnaire that imepitoin had a greater reduction on fear/anxiety during storms (P = 0.0005), and during other noise events (P = 0.0001) than placebo. 78.6% (22/28) of owners in the active imepitoin group agreed that treatment reduced their dog's signs of fear and anxiety during storms, compared with 26.7% (4/15) of owners in the placebo group. 80% (24/30) of the imepitoin group experienced adverse events compared with 13% (2/15) of the placebo group, most frequently ataxia 60.0% (18/30) imepitoin vs 6.7% (1/15) placebo and increased appetite 30.0% (9/30) imepitoin vs 6.7% (1/15) placebo.
Limitations:	No power calculation performed for optimal group size, although with a small population size, it is likely to be underpowered. Selection bias resulting from convenience sample recruited through flyers, social media, or local vets. No indication whether dogs were screened for pain as a confounding factor. No mention of screening for thyroid disease during blood tests. Proportionally more female dogs than male dogs in placebo group (3 males; 12 females) compared to active group (15 males; 15 females) (P = 0.06). Randomisation procedure not detailed. Reasons for 2:1 randomisation not given. Constituents of placebo not detailed. Subjective assessment of storm intensity and behaviours by owners. No expert behaviour coding or measurements of physiological markers of fear/stress response. Duration and intensity of storms, which may have affected results, not included in analysis. Reliance on accurate dosing and reporting by owners, and no aspects of the dogs' home environment, including owners' response to their dog's behaviour, were controlled for. No details given of other ‘noise events’ examined in final questionnaire. Amount of imputed data in intention-to-treat group not quantified. No numerical data or p-value for storm log scores, wide 95% confidence interval for storm log scores [–∞, –0.03813] Hard to determine actual LSSS intensity scores from plots of estimated lines for storm log scores. High numbers of adverse events in active group (24/30 compared to 2/15 in placebo group) could result in unblinding and risks bias in the results. High withdrawal rate post randomisation (10/30 in active group) potentially affects validity of per-protocol results. LSSS scores of dropouts not analysed, potentially lower treatment group scores may be due to loss of subjects with high LSSS scores. Possible caregiver placebo effect resulting in improvements in both group’s scores. In placebo group 4/15 (26.7%) owners agreed that treatment reduced fear or anxiety during storms. Intra-rater reliability of LSSS unknown, so possible confounding effect of individual owners using LSSS repeatedly in this study. Study funded by, and one co-author was an employee of Boehringer Ingelheim manufacturers of Pexion® (imepitoin).

### Appraisal, application and reflection

An excessive fear of loud noises is a common problem in the UK canine population with between 25–49% of dogs affected; despite this, many owners are unaware of the treatment options available (Blackwell et al., 2013). Desensitising programmes utilising recordings of fear-eliciting sounds, alongside short-term management changes, are recommended to manage noise sensitivity (Sherman & Mills, 2008). These programmes should be started a minimum of 60 days in advance of anticipated events and accompanying instructions followed carefully (Levine et al., 2007) which in practice, is not always possible for some owners. Short-term psychopharmaceutical intervention for distressed dogs is therefore warranted on welfare grounds. Imepitoin is one of the three licensed psychopharmaceuticals available.

Two papers were considered suitable for appraisal, both randomised placebo-controlled trials (Engel et al., 2019; Perdew et al., 2021). Notably, both studies were funded by Boehringer Ingelheim, who manufacture the veterinary licensed form of imepitoin. The study by Engel et al. (2019) directly assessing fear and anxiety caused by fireworks had the larger sample size of the two studies appraised. Results were given for the full analysis set (FAS)  226 dogs and included 18 dogs who had less than the full number of treatment doses and one dog in the placebo group who received concomitant treatment. This may, however, reflect nonexperimental scenarios where compliance is not always perfect. The study reported that fear and anxiety behaviours were statistically significantly reduced in the imepitoin group versus placebo group at all time points. Additionally, behaviours indicating fear or anxiety were reduced in dogs given imepitoin versus placebo, which was concluded in a later study to be a clinically relevant difference (Mills et al., 2020). Significantly lower mean modified Lincoln Sound Sensitivity Scale (LSSS) scores (Mills et al., 2012) were seen in the imepitoin group in the Engel et al. (2019) study at maximum firework intensity. Their explanatory box and whisker plot, however, illustrates the variability of response with some dogs responding better than others. The modified LSSS questionnaire used in both studies aims to increase the objectivity of the data collected (Mills et al., 2012) and appeared to be reliable when it was initially validated during the Engel et al. (2019) study (Mills et al., 2020).

The paper by Perdew et al. (2021) examined client-owned dogs with fear and anxiety elicited by storms. Whilst the sound of fireworks most commonly triggers behavioural fear reactions, it has been suggested that dogs that have a phobia of fireworks are highly likely to also display a fear of gunshots and thunder (Blackwell et al., 2013; Riemer, 2019). Perdew et al. (2021) reported a reduction in weekly survey scores (assessment of all storms over the previous week) from baseline in dogs receiving imepitoin. A reduction was also seen in the placebo group with 26.7% of owners (compared to 78.6% in the imepitoin group) agreeing that the treatment reduced their dog’s signs of fear and anxiety. This percentage is similar to the approximately 25% of owners in the placebo group who rated treatment as ‘excellent’ or ‘good’ in the study by Engel et al. (2019), suggesting a caregiver placebo effect. Perdew et al. (2021) also reported significant differences between storm log scores for imepitoin and placebo groups. However, no figures (other than a line graph) or  p-value are included in the results, and the wide 95% confidence interval of –∞ to –0.03813 reflects the small sample size and uncertainty about any conclusions drawn.

Both studies reported high incidences of mild-to-moderate adverse events (side effects) particularly in the first week. In Engel et al. (2019) 48% of the imepitoin group compared to 10% in the placebo group had side effects including 35% of dogs developing ataxia, although the ataxia was reported to resolve in three quarters of cases over the 24 to 48 hours following treatment inception. Whereas Perdew et al. (2021) reported that 80% of the imepitoin group compared to 13% in the placebo group experienced adverse events including ataxia, appetite changes, and somnolence. As a result, 10/30 (33.3%) dogs taking imepitoin in the Perdew et al. (2021) study withdrew. Such a high number of dogs lost potentially invalidates any benefit of randomisation as participants are not lost equally and randomly from both groups, introducing bias into the per protocol analysis (Lachin, 2000). Similarly, the necessity to impute data for an undisclosed proportion of the imepitoin group could also affect the validity of the intention-to-treat results (Streiner & Geddes, 2001). In addition, 90% of owners in the Perdew et al. (2021) imepitoin group correctly guessed what group their dog was in, suggesting possible unblinding (Forbes, 2013) potentially affecting the validity of results which relied upon owner report.

The potential distress caused to owners by observing signs of fear and anxiety in their dogs responding to loud noises may have affected the ability of some of the owners in these studies to accurately record their dog’s behaviour. Scoring of owner-recorded videos by canine behaviour experts could have been used to detect signs of noise phobia potentially missed by owners (Van Belle et al., 2023). However, it is important to note that even with good inter- and intra-observer reliability, adequate blinding of the video assessors may have been difficult given the degree of noticeable side effects seen in the imepitoin treatment groups (Tuyttens et al., 2014).

The rationale for Engel et al. (2019) choosing a dose of 30 mg/kg twice daily was to rapidly establish an efficacious serum imepitoin concentration and based in part on a case series by McPeake & Mills (2017) which included 14 dogs with unspecified noise sensitivities. Out of those 14 dogs, only five were on a dose of around 30 mg/kg twice daily at the final follow up after 11 to 19 weeks. McPeake & Mills (2017) proposed that for dogs diagnosed with fear-anxiety disorders doses of 20 mg/kg imepitoin twice daily may be sufficient for anxiolysis. Indeed Engel et al. (2019) lowered daily doses of medication to mitigate ataxia in seven cases (five in imepitoin group, two in placebo group) although when and by how much was not described.

Whilst not directly related to the PICO question, the potential side effects of imepitoin are of note with respect to an owner’s willingness to continue the treatment, and to the possible impact that being ataxic or vomiting may have on a dog already experiencing an aversive event. Trialling lower doses for efficacy without side effects before titrating upwards could therefore be considered an appropriate approach.

Furthermore, a small pilot study with no placebo control by Muñoz Amezcua et al. (2024) examined an individualised dose titration approach for imepitoin, reporting a reduction in signs of anxiety during storms using doses ranging from 10 to 30 mg/kg twice daily. During the 12-week study period, the initial dose of 10mg/kg twice daily was increased every two weeks if there was a perceived lack of response to 20mg/kg twice daily or 30mg/kg twice daily.  Whilst lower doses were effective for some dogs, an increase in dose was required for many others. By the end of the study, 1/33 dogs remained on the 10mg/kg dose, 3/33 on the 20mg/kg dose and 11/33 on the 30mg/kg dose. The remaining dogs withdrew due to a perceived lack of effect or adverse effects. As in the appraised studies, the rate of mild-to-moderate adverse events including ataxia, appetite changes, and ‘irritability/aggressive behaviour’ was high and was notably more prevalent in dogs receiving 20 mg/kg imepitoin twice daily. Further large-scale trials are needed to confirm whether doses lower than that recommended by the manufacturer can maintain therapeutic effect, whilst minimising side effects.

Imepitoin is one of three drugs currently licensed and available for treating noise phobias in the UK and should be considered in preference to unlicensed medication. The evidence for using imepitoin is considered moderate due to limitations including a low number of randomised placebo-controlled studies, variability of results, subjective assessment bias, and high incidence of side effects potentially affecting the validity of results. Clients need to be made aware of any possible side effects and that treatment outcomes may vary, necessitating dose titration for some dogs. Any dose alterations or treatment failures should be recorded in clinical records in such a way that their prevalence can be monitored within a practice setting, and discussions had amongst clinicians about whether for example a different starting dose to that recommended should be used.

### Methodology

**Search Strategy:** 

Databases searched and dates covered:	CAB Abstracts on OVID platform: 1973 to Week 27 2025 Scopus on Elsevier: 1995 to 2025 Web of Science Core Collection on Clarivate: 1900 to 2025 PubMed on National Library of Medicine (USA): 1946 to 2025
Search strategy:	CAB Abstracts: (dog* or canine* or bitch* or canis or canid*).mp. or exp dogs/ or exp canis/ (imepitoin or Pexion or "ELB 138" or "AWD 131-138").mp. (sound* or storm* or thunderstorm* or thunder*or noise* or firework* or shot* or shoot*).mp. (Fear* or fearful* or phobi* or aversive or aversion or anxiety or anxio* or nervous or fright* or sensitiv*).mp. or exp anxiety/ or exp emotions/ or exp anxiolytic properties/or exp mental stress/ or exp phobias/ 1 and 2 and 3 and 4 Scopus: (TITLE-ABS-KEY (dog* OR canine* OR bitch* OR canis OR canid*) AND TITLE-ABS-KEY (sound* OR storm* OR thunderstorm* OR thunder* OR noise* OR firework* OR shoot* OR shot*) AND TITLE-ABS-KEY (fear* OR phobi* OR aversiv* OR aversion OR anxiety OR anxio* OR nervous OR fright*) AND TITLE-ABS-KEY (imepitoin OR pexion OR “ELB 138” OR “AWD 131-138”)) Web of Science Core Collection: Dog* OR canine* OR bitch* OR canis OR canid*(All fields) and sound* OR storm* OR thunderstorm* OR thunder* OR noise* OR firework*OR shot* OR shoot* (All fields) and imepitoin OR Pexion OR "ELB 138" OR "AWD 131-138" (All fields) and Fear* OR fearful* OR phobi* OR aversiv* OR aversion OR anxiety OR anxio* OR nervous OR fright*OR sensitiv*(All fields) PubMed: ((Dog OR canine* OR bitch* OR canis OR canid* AND (english[Filter])) AND (Sound* OR storm* OR thunderstorm* OR thunder* OR noise* OR firework* OR shot* OR shoot* AND (english[Filter]))) AND (imepitoin OR Pexion OR ELB 138 OR AWD 131-138 AND (english[Filter]))) AND (fear OR fearful* OR phobi* OR aversiv* OR aversion OR anxiety OR anxio* OR nervous OR fright* OR sensitiv* AND (english[Filter]))
Dates searches performed:	07 July 2025

**Exclusion / Inclusion Criteria:** 

Exclusion:	Not in English language. Irrelevant to PICO. Case series or case reports. Conference proceedings. Opinion or review articles.
Inclusion:	Peer-reviewed articles.

**Search Outcome:** 

Database	Number of results	Excluded – not in English	Excluded – did not answer the PICO	Excluded – opinion piece/review article	Excluded – case series/reports	Excluded – unable to access	Total relevant papers
CAB Abstracts	12	3	2	2	1	2	2
Scopus	10	2	3	2	1	0	2
Web of Science	8	2	3	0	1	0	2
PubMed	6	0	3	0	1	0	2
Total relevant papers when duplicates removed							2

## Acknowledgements

The author would like to thank Maureen O’Mara for her advice and support.

## ORCiD

Hannah McNicholas: 0009 0006 3695 3796

## Disclaimer

Knowledge Summaries are a peer-reviewed article type which aims to answer a clinical question based on the best available current evidence. It does not override the responsibility of the practitioner. Informed decisions should be made by considering such factors as individual clinical expertise and judgement along with patient’s circumstances and owners’ values. Knowledge Summaries are a resource to help inform and any opinions expressed within the Knowledge Summaries are the author's own and do not necessarily reflect the view of the RCVS Knowledge. Authors are responsible for the accuracy of the content. While the Editor and Publisher believe that all content herein are in accord with current recommendations and practice at the time of publication, they accept no legal responsibility for any errors or omissions, and make no warranty, express or implied, with respect to material contained within. For further information please refer to our Terms of Use.
